# Benchmarking of feed-forward neural network models for genomic prediction of quantitative traits in pigs

**DOI:** 10.3389/fgene.2025.1618891

**Published:** 2025-06-18

**Authors:** Junjian Wang, Francesco Tiezzi, Yijian Huang, Christian Maltecca, Jicai Jiang

**Affiliations:** ^1^ Department of Animal Science, North Carolina State University, Raleigh, NC, United States; ^2^ Department of Agriculture, Food, Environment and Forestry (DAGRI), University of Florence, Florence, Italy; ^3^ Smithfield Premium Genetics, Rose Hill, NC, United States

**Keywords:** deep learning, genomic prediction, duroc pigs, complex traits, predictive ability

## Abstract

Artificial neural networks are machine learning models that have been applied to various genomic problems, with the ability to learn non-linear relationships and model high-dimensional data. These advanced modeling capabilities make them promising candidates for genomic prediction by potentially capturing the intricate relationships between genetic variants and phenotypes. Despite these theoretical advantages, neural networks have shown inconsistent performance across previous genomic prediction research, and limited studies have evaluated their performance and feasibility specifically for pig genomic predictions using large-scale data. We evaluated the predictive performance of feed-forward neural network (FFNN) models implemented in TensorFlow with architectures ranging from single-layer (no hidden layers) to four-layer structures (three hidden layers). These FFNN models were compared with five linear methods, including GBLUP, LDAK-BOLT, BayesR, SLEMM-WW, and scikit-learn’s ridge regression. The evaluation utilized data from six quantitative traits: off-test body weight (WT), off-test back fat thickness (BF), off-test loin muscle depth (MS), number of piglets born alive (NBA), number of piglets born dead (NBD), and number of piglets weaned (NW). We also assessed the computational efficiency of FFNN models on both CPU and GPU. The benchmarking employed repeated random subsampling validation with sample sizes ranging from 3,290 individuals for reproductive traits to over 26,000 individuals for production traits, using data from a total of 27,481 genotyped pigs. Hyperband tuning was used to optimize the hyper-parameters and select the best model for each structure. Results showed that FFNN models consistently underperformed compared to linear methods across all architectures tested. The one-layer structure yielded the best predictive accuracy among the FFNN approaches. Of the five linear methods, SLEMM-WW demonstrated the best balance of computational efficiency and predictive ability. GPUs offered significant computational efficiency gains for multi-layer FFNN models compared to CPUs, though FFNN models remained more computationally demanding than most linear methods. In conclusion, FFNN models with up to four layers did not improve genomic predictions compared to routine linear methods for pig quantitative traits.

## Introduction

Selective breeding has long been fundamental to improving livestock traits since the early 20th century and guided by quantitative genetics theory established by Sir Ronald Fisher’s infinitesimal model (Fisher, 1919). With the availability of cost-effective SNP chips, genomic prediction, which refers to the use of genome-wide DNA markers to estimate the breeding values has been widely adopted in animal breeding programs, enabling early and accurate selection of candidates to accelerate genetic progress (Meuwissen et al., 2001).

The effectiveness of genomic selection depends on statistical models that relate high-dimensional genotype data to phenotypes. Traditional genomic prediction methods predominantly rely on linear mixed models that estimate the contribution of genetic markers under the assumption of additive genetic effects. One of the most commonly used methods is Genomic Best Linear Unbiased Prediction (GBLUP), which assumes marker effects are random draws from a multivariate normal distribution with a common variance component (Meuwissen et al., 2001; VanRaden, 2008). However, the assumption of identical variance for all SNP effects can be limiting when the genetic architecture includes large-effect quantitative trait locus (QTLs) or major genes (Daetwyler et al., 2010; Resende et al., 2012; de los Campos et al., 2013). Therefore, various Bayesian methods have been developed that allow for more flexible prior distributions of SNP effects. Bayesian methods like BayesA, BayesB, BayesCπ, and BayesR generally outperform GBLUP when a few loci have large effect for a trait (Clark et al., 2011; Resende et al., 2012; Moser et al., 2015; van den Berg et al., 2015). These methods typically incur high computational costs, as they often rely on intensive Markov chain Monte Carlo sampling. Recent approaches such as LDAK (Linkage Disequilibrium Adjusted Kinships) and SLEMM (Stochastic Lanczos Expedited Mixed Models) employ faster algorithms and optimized strategies to reduce computation time while maintaining high prediction accuracy. While traditional genomic prediction methods have proven effective, they predominantly rely on linear mapping from genotype to phenotype under additive genetic assumptions, potentially limiting their ability to capture non-linear genetic architectures. With improvements in computational hardware, particularly graphics processing units (GPUs), artificial neural networks (ANNs) have received increased attention (Raina et al., 2009). ANNs are a class of machine learning methods inspired by the biological structure and function of the human brain, comprising interconnected units that simulate the behavior of neurons (McCulloch and Pitts, 1943; Newell and Simon, 2007). Each connection, like the synapses in a biological brain, can transmit a signal to other neurons. Complex neural networks characterized by multiple hidden layers form the cornerstone of deep learning (DL). A key theoretical advantage of neural networks in genomic prediction is their capability as universal function approximators (Kůrková, 1992). Unlike conventional linear methods, deep learning approaches are nonparametric models that provide tremendous flexibility to adapt to complex relationships between inputs and outputs. Given sufficient data and appropriate architectures, they can model complex relationships without requiring explicit specification of interactions beforehand (Montesinos-López et al., 2021). This capability suggests potential advantages over linear methods, particularly for traits influenced by dominant, epistatic, or other non-additive genetic effects (Zingaretti et al., 2020; Costa et al., 2022). By learning directly from the data, neural networks might capture complex genetic architectures that traditional parametric linear models cannot easily accommodate, potentially improving prediction accuracy for traits with complex inheritance patterns.

Due to the ability to handle large datasets and capture complex relationships, artificial neural network algorithms have significantly advanced data science methodologies and have made multiple performance breakthroughs in computer vision, speech recognition, video processing, and natural language processing applications (LeCun et al., 2015). Their remarkable performance in these areas has motivated researchers to explore their potential application for genomic prediction in humans, livestock, and plants. However, despite their theoretical promise, most applications in livestock genomic prediction have not outperformed conventional linear methods, with several studies reporting comparable or decreased performance (Bellot et al., 2018; Montesinos-López et al., 2018; Azodi et al., 2019; Zingaretti et al., 2020; Srivastava et al., 2021; Lourenço et al., 2024). Conversely, some investigations have reported modest improvements in prediction accuracy for specific traits (Gianola et al., 2011; Ma et al., 2018; Waldmann, 2018; Liu et al., 2019; 2025; Abdollahi-Arpanahi et al., 2020; Sandhu et al., 2021). These inconsistent findings may stem from differences in species, traits, reference population size, marker density, or neural network architecture.

In pig breeding, genomic prediction has been implemented since the early 2010s (Christensen et al., 2012). The primary breeding objectives focus on growth performance traits (average daily gain, feed efficiency), carcass composition traits (backfat thickness, loin muscle area), and reproductive performance traits (litter size, number born alive), which collectively determine the economic viability of pig production systems (Knol et al., 2016; Samorè and and Fontanesi, 2016). More recently, welfare and resilience traits such as heat stress tolerance (Freitas et al., 2023; Wen et al., 2024) and disease resistance (Bai and Plastow, 2022; Cheng et al., 2022) have been considered for incorporation to address climate change and sustainability challenges.

Currently, comprehensive evaluations of neural networks for genomic prediction in pigs remain limited, necessitating systematic benchmarking against conventional linear methods to assess their practical utility in pig breeding programs. Among various neural network architectures, feed-forward neural networks (FFNNs) represent one of the most fundamental and widely applied approaches in deep learning (Goodfellow, 2016). Their ability to approximate complex functions while maintaining relative simplicity makes them a logical starting point for evaluating the potential of neural networks in genomic prediction applications.

The computational demands of neural networks present another important consideration for their implementation in routine genomic evaluations. Traditional linear methods have been optimized for computational efficiency, enabling rapid evaluation of large populations. In contrast, neural networks, particularly those with complex architectures, typically require substantial computational resources for hyper-parameter tuning, model training, and implementation. Training deep learning models on large genomic datasets can be time-consuming and may require specialized hardware such as GPUs or TPUs to reduce training times and facilitate large-scale analyses (O’Connell et al., 2023; Schmidt and Hildebrandt, 2024). Therefore, it is important to investigate the practical computational efficiency of neural networks with varying complexities when evaluating the feasibility of neural networks for integration into practical breeding applications.

The objective of this study is to comprehensively evaluate FFNN models with varying architectural complexity for genomic prediction in pigs. Specifically, we assessed the predictive performance of FFNNs relative to conventional linear methods for complex traits, determined the computational efficiency of all approaches on CPU platforms, and evaluated the potential acceleration of FFNN models through GPU implementation. This benchmarking provides valuable insights into the practical utility of FFNN models in pig breeding programs.

## Materials and methods

### Duroc pig datasets

This study utilized phenotypic and genotypic data from Duroc pigs collected by Smithfield Premium Genetics between 2015 and 2021. Six complex traits of Duroc pigs, including three performance traits and three reproduction traits, were used for analysis, with specific sample sizes and heritability for each trait shown in Table 1. The three performance traits were collected at the end of the performance test, as detailed in Bergamaschi et al. (2020). In total, 27,481 pigs with both valid genotypes and phenotypes were retained for the analysis.

**TABLE 1:** Trait descriptions, sample sizes, and heritability estimates for six complex traits in Duroc pigs.

Trait name	Abbreviation	Number of individuals	Heritability
Off-test Back fat thickness	BF	26,731	0.371
Off-test Loin muscle depth	MS	26,731	0.205
Off-test body weight	WT	27,360	0.103
Number of piglets born alive	NBA	3,290	0.096
Number of piglets born dead	NBD	3,290	0.051
Number of piglets weaned	NW	3,290	0.062

Phenotypes for all traits were pre-adjusted to account for systematic environmental effects. The adjusted phenotypic values only included genetic and environmental variation proper of the individual, being adjusted for farm and physiological sources of variation. All pre-adjusted phenotypes were then standardized prior to implementing the genomic prediction models. All animals were genotyped with PorcineSNP60 BeadChip (Illumina Inc., San Diego, CA, United States). Genotype quality control was performed using Plink1.90. SNPs were filtered based on Hardy-Weinberg equilibrium (HWE) P-value > 1E-8 and minor allele frequency (MAF) > 0.01. After quality control, 30,981 SNPs remained for subsequent analysis.

### Artificial neural network models

Feed-forward neural networks (FFNNs) represent a class of artificial neural networks characterized by unidirectional information flow, where connections between nodes do not form cycles. In this study, we implemented fully connected FFNNs, where each neuron in a layer is connected to every neuron in the subsequent layer. It has three types of layers: input layer, hidden layers and output layer. The input layer receives data, hidden layers process this information through non-linear transformations, and the output layer produces the final predictions. When an FFNN contains at least one hidden layer, it is also referred to as a multilayer perceptron (MLP).

**FIGURE 1:**
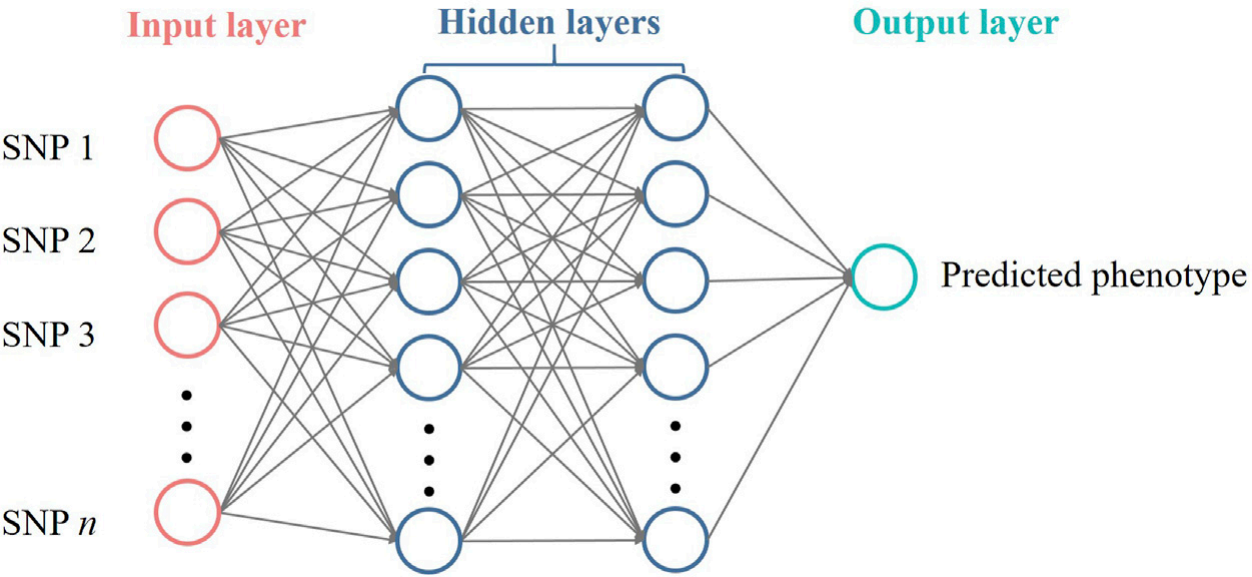
Architecture of a three-layer FFNN (with two hidden layers) for genomic prediction. The network architecture illustrates the flow of information from input to output. The input layer receives SNP genotype data (coded as 0, 1, or 2), the hidden layers process this information through non-linear transformations, and the output layer produces the predicted phenotypic value. Each circle represents a neuron, and connections between neurons represent weights that are optimized during model training.

Figure 1 illustrates a fully connected FFNN architecture for genomic prediction, comprising an input layer with *n* SNP markers, two hidden layers with multiple neurons, and an output layer with a single neuron. For genomic prediction purposes, the input layer receives SNP genotype data, and information propagates sequentially through the hidden layers as weighted non-linear transformations. Each hidden neuron computes a weighted sum of its inputs plus a bias term, applies a non-linear activation function to this sum, and passes the resulting value to neurons in the subsequent layer. The output neuron aggregates the signals from the previous layer to produce a predicted phenotype.

We implemented FFNN models using TensorFlow v.2.6.2 (Abadi et al., 2015). The architectures evaluated consisted of one to four layers in total, corresponding to models with no hidden layers (one-layer networks), one hidden layer (two-layer networks), two hidden layers (three-layer networks), and three hidden layers (four-layer networks). For each model, the input layer received the normalized SNP data, while the output layer produced continuous predictions for each trait. Hidden layers employed the non-linear activation function to capture the complex relationships, and the output layer used a linear activation function. An *L*-layer structure FFNN model with (*L*-1) hidden layers can be expressed as:

$$a^{\left[0\right]}=X$$

$$a^{\left[l\right]}=\sigma^{\left[l\right]}\left(W^{\left[l\right]}a^{\left[l-1\right]}+b^{\left[l\right]}\right)\text{ for }l=1,2,\cdots ,L-1$$

$$\hat{y}=W^{\left[L\right]}a^{\left[L-1\right]}+b^{\left[L\right]}$$

where $a^{\left[0\right]}$ is the input feature derived from the normalized genotype matrix **X** as input, with genotypes coded as 0, 1, and 2 for the homozygote for the major allele, heterozygote, and homozygote for the minor allele, respectively, and then normalized. $a^{\left[l\right]}$ represents the activations for layer *l*, $\sigma^{\left[l\right]}$ is the activation function applied at the *l*-th hidden layer, $W^{\left[l\right]}$ and $b^{\left[l\right]}$ are the weight matrix and bias vector for layer *l*, respectively, *L* denotes the total number of layers including hidden layers and the output layer, and $\hat{y}$ is the predicted phenotype.

### Hyper-parameter optimization

Hyper-parameters are model configuration settings that govern the learning process and network architecture. Unlike model parameters (such as weights and biases in a neural network), which are learned directly from the training data, they are not updated during training, but must be specified prior to training and remain fixed throughout.

For optimal model performance, we conducted systematic hyper-parameter tuning using the Hyperband algorithm implemented in Keras Tuner (https://keras.io/keras_tuner/api/tuners/). Hyperband functions as an efficient search methodology that balances breadth and depth of exploration (Li et al., 2018). It accelerates the traditional hyper-parameter optimization process by evaluating many configurations on a small subset of data and iterations, then allocating additional computational resources only to the most promising configurations. This method provides an efficient alternative to exhaustive grid search or random search approaches, particularly for computationally intensive neural network training.

**FIGURE 2:**
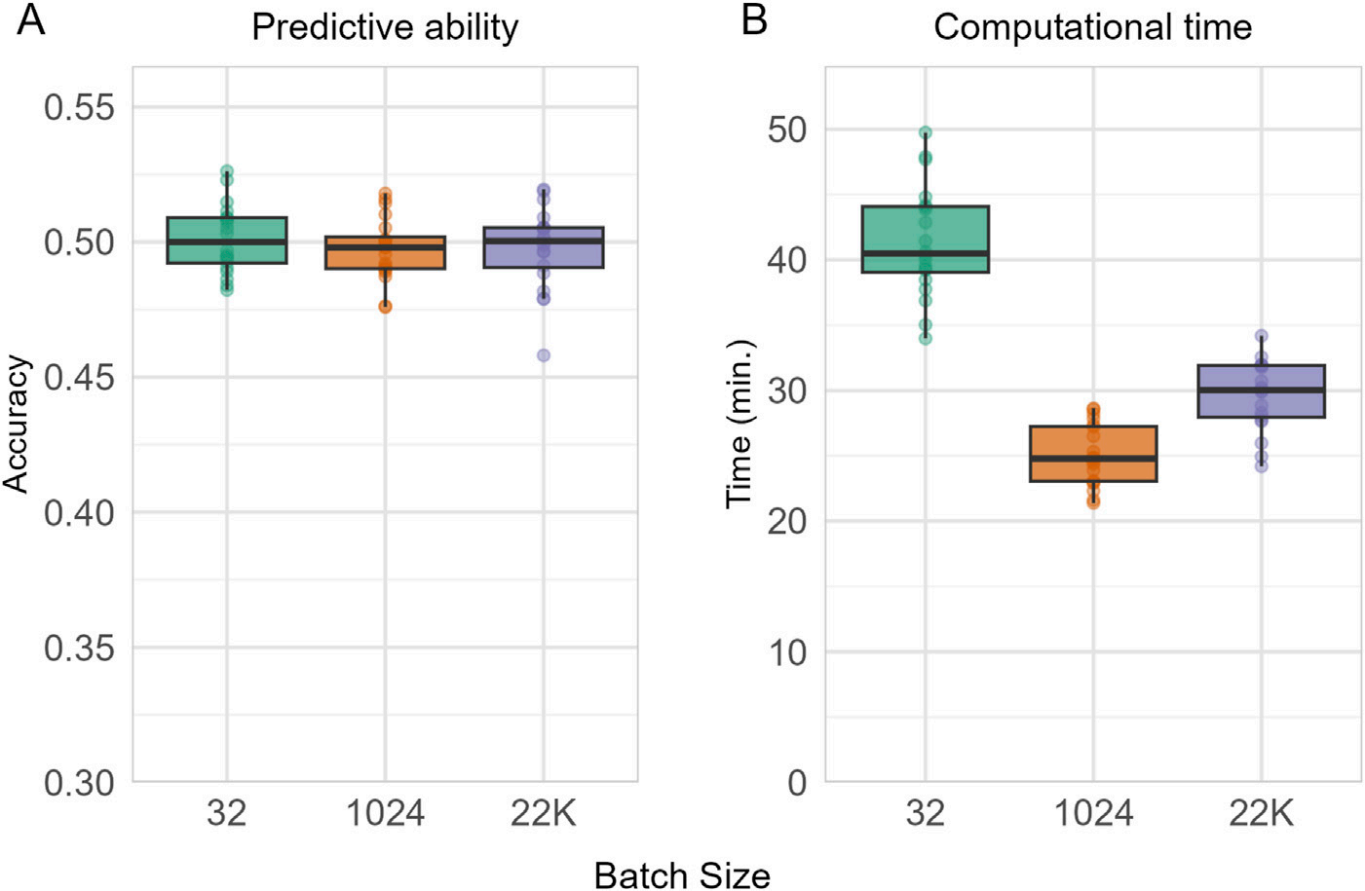
Comparison of **(A)** predictive ability (measured by Pearson correlation coefficient) and **(B)** computational time across different batch sizes (32, 1024, and 22k) for one-layer FFNN model tested on BF. Results show the distribution across 20 random subsampling replicates, with 21,385 individuals for training and 5,346 for testing.

We optimized five key hyper-parameter categories: network capacity (number of units per layer), non-linear transformations (activation functions), regularization methods (L2 penalty and dropout rate), optimization strategy (learning rate), and training duration (epochs). The complete search space for FFNN hyper-parameters is defined in Table 2. To prevent overfitting during the hyper-parameter search process, we implemented early stopping with a patience of 20 epochs. A batch size of 1024 was selected based on preliminary experiments with one-layer FFNN models for BF trait prediction across 20 replicates. We tested three batch sizes (32, 1024, and 22K) and found that while all three provided similar predictive ability, the 1024 batch size offered better computational efficiency (Figure 2). All model configurations were validated on a randomly selected 20% holdout portion of the training data. This internal validation set allowed for model performance monitoring and early stopping to prevent overfitting. The best model with the optimal hyper-parameters was then retrained for the following genomic prediction.

**TABLE 2:** Hyper-parameter search space for FFNN models.

Hyper-parameters	Search range	Sampling method
Number of units	32–1024 (step of 32)	Uniform discrete
Activation function	ReLU, sigmoid, tanh, softmax	Categorical
L2 regularization	0.01–20	Logarithmic
Dropout rate	0.0, 0.01, 0.02, 0.04, 0.06, 0.08, 0.1, 0.2, 0.3, 0.4, 0.5	Categorical
Learning rate	1E-3, 1E-4, 1E-5	Categorical
Number of epochs	≤1000 (early stopping, patience = 20)	—

### Conventional statistical methods

To evaluate the predictive performance of FFNN for genomic prediction in pigs, we conducted comprehensive benchmarking against five commonly used linear methods including genomic best linear unbiased prediction (GBLUP), BayesR (Moser et al., 2015), scikit-learn’s ridge regression (Pedregosa et al., 2011), LDAK’s BOLT method (LDAK-BOLT) (Zhang et al., 2021) and SLEMM window-weighted method (SLEMM-WW) (Cheng et al., 2023). These methods share similar statistical frameworks based on linear relationships between genetic markers and phenotypic traits, but differ in their assumptions about genetic architecture and employ various computational strategies to improve efficiency and accuracy. The comparison allowed us to assess whether the potential non-linear modeling capabilities of neural networks provided advantages over conventional approaches.

GBLUP is one of the most extensively used linear regression methods for genomic prediction and implements a mixed linear model approach using a genomic relationship matrix to capture genetic similarities between individuals (VanRaden, 2008). In this study, it was implemented with SLEMM v0.90.1 and assumes that all SNPs contribute equally to SNP heritability.

BayesR extends the linear mixed model framework through a Bayesian approach that allows SNP effects to follow a mixture of four normal distributions, with variance components set to 0, 0.0001, 0.001, and 0.01 of the genetic variance. This approach was implemented with BayesRv2 (Moser et al., 2015), which employs a computationally optimized Gibbs sampling algorithm to effectively accommodate complex genetic architectures characterized by varying SNP effect sizes. We set blocksize = 4, msize = 500, number of MCMC iterations = 50,000, and burn-in = 20,000 iterations in this study.

Ridge regression applies L2 regularization to the standard linear regression model, penalizing large coefficient values to prevent overfitting in high-dimensional genomic data. This method is mathematically equivalent to GBLUP under specific parameterizations, when the ridge regularization parameter λ equals ${{\;\sigma }_{e}}^{2}/{\sigma_{g}}^{2}\times m$ , where ${\sigma_{e}}^{2}$ and ${\sigma_{g}}^{2}$ are the residual and genetic variances, respectively, and *m* is the number of markers (Endelman, 2011). We included ridge regression as implemented in scikit-learn to evaluate computational efficiency using different optimization strategies compared to GBLUP. Since the optimal regularization strength was unknown *a priori* and this parameter is related to trait heritability, we determined appropriate λ values based on previous reported heritability estimates for each trait. For back fat thickness, previous research indicates heritability ranging from 0.3 to 0.58 (Ros-Freixedes et al., 2013; Guo et al., 2016; Davoli et al., 2019; Gozalo-Marcilla et al., 2021). We conducted a grid search across λ values corresponding to heritabilities ranging from 0.3 to 0.6 with increments of 0.01. For each potential λ value, we performed five-fold cross-validation to identify the parameter that minimized the mean squared error.

LDAK-BOLT combines the LDAK framework with a Bayesian approach similar to BOLT-LMM (Loh et al., 2015), which models SNP effects using a mixture of two zero-mean normal distributions, one with a relatively small variance and the other with a relatively large variance. This method offers computational efficiency for large-scale genomic data while accommodating markers of varying effect sizes. In our implementation we used equal weights for all SNPs and applied default parameters using LDAK v5.2.

SLEMM-WW is a linear mixed model-based genomic prediction method incorporating a window-based weighting strategy. The method employs a two-step process: first fitting an initial model assigning equal weights to all SNPs, then refitting with weights calculated from neighboring SNP effects within a fixed window. This weighting scheme leverages the assumption that SNPs close to each other share similar effects due to their proximity to underlying quantitative trait loci. In this study we implemented it with SLEMM v0.90.1 using the default fixed window size of 20. This method has demonstrated ability to improve prediction accuracy while maintaining computational efficiency in large-scale genomic prediction applications.

### Repeated random subsampling validation

We used repeated random subsampling validation with 20 iterations to evaluate the predictive ability of the aforementioned methods with the six complex traits. For each iteration, identical random partitions were applied to all methods, ensuring fair comparison under the same validation conditions. The dataset was randomly partitioned into a training population (about 80% of individuals) and a test population (about 20% of individuals). For FFNN models, the training population was further divided during model fitting, with 80% used for actual training (64% of total individuals) and 20% used for validation (16% of total individuals) to monitor model performance and prevent overfitting during the training process. Predictive ability was assessed using the Pearson correlation coefficient between predicted values and observed phenotypes in the test population.

To evaluate computational speed, all analyses were initially conducted on a Linux server equipped with two Intel Xeon Gold 6258R CPUs. Twenty threads were used for scikit-learn’s ridge regression, SLEMM-GBLUP, SLEMM-WW, LDAK-BOLT and FFNN models, whereas four threads (equal to block size) were used for BayesR, since using twenty threads would have considerably slowed down its computations. To comprehensively assess the computational efficiency of neural network approaches across different hardware platforms, we also evaluated FFNN models on three GPU configurations: NVIDIA A30, NVIDIA RTX 3060 Ti, and NVIDIA RTX 3090. This multi-platform evaluation allowed us to quantify the potential acceleration of genomic prediction calculations through specialized hardware.

## Results

Prior to the main analysis, we conducted preliminary experiments to determine the optimal batch size for FFNN training. Using the one-layer FFNN model for BF trait prediction across 20 replicates, we evaluated three batch sizes: 32 (small), 1024 (medium), and 22K (large, approximately the full training set). Predictive ability was measured as the Pearson correlation coefficient between observed and predicted phenotypic values. While all three batch sizes yielded similar predictive accuracy, significant differences were observed in computational efficiency (Figure 2), with the medium batch size of 1024 providing the best performance. Therefore, a batch size of 1024 was used for all subsequent FFNN analyses.

### Predictive ability

**FIGURE 3:**
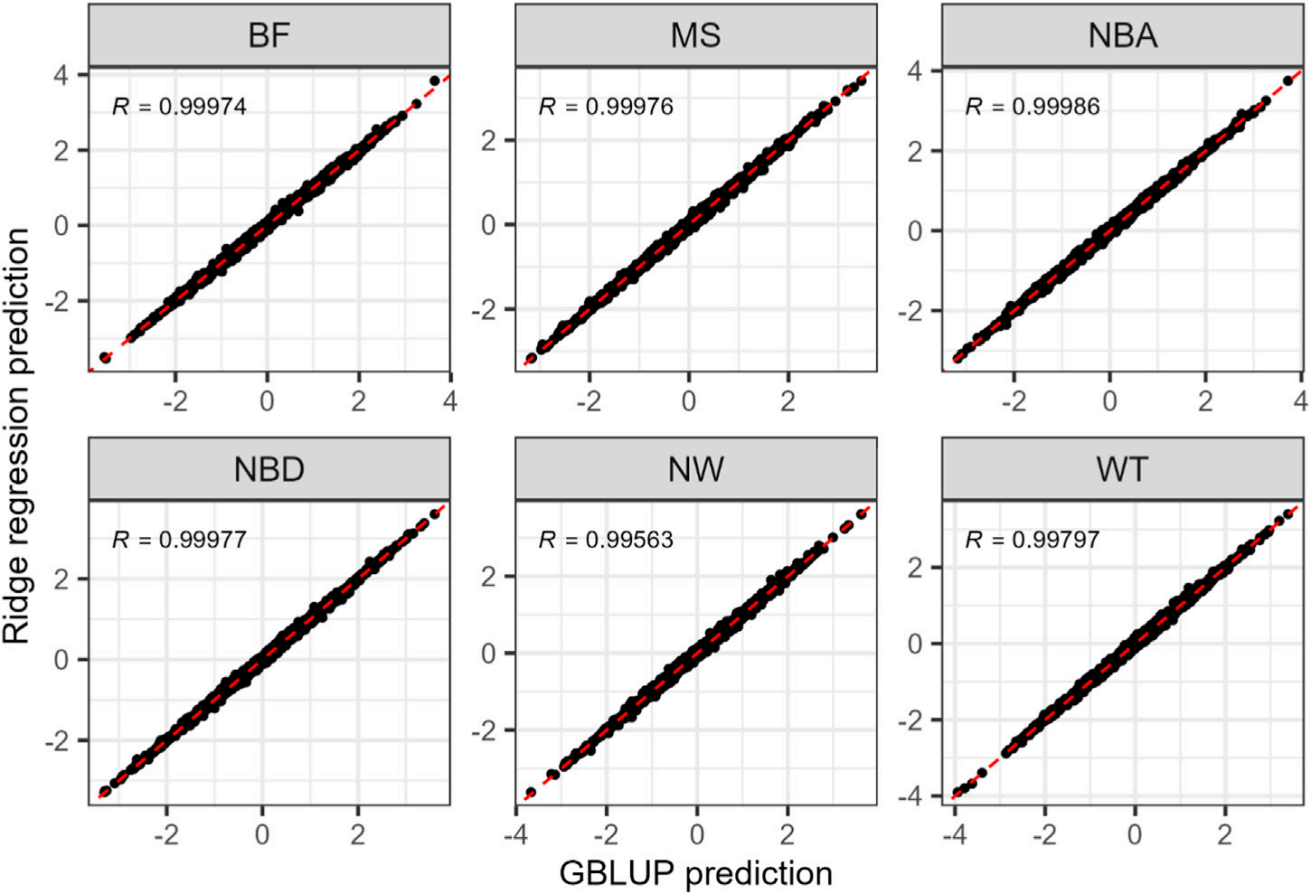
Comparison of standardized predictions from GBLUP and ridge regression across six traits in one replicate. Each point represents an individual, and the dashed line indicates the identity line (y = x). Pearson correlation coefficients (*R*) are shown for each trait panel.

**FIGURE 4:**
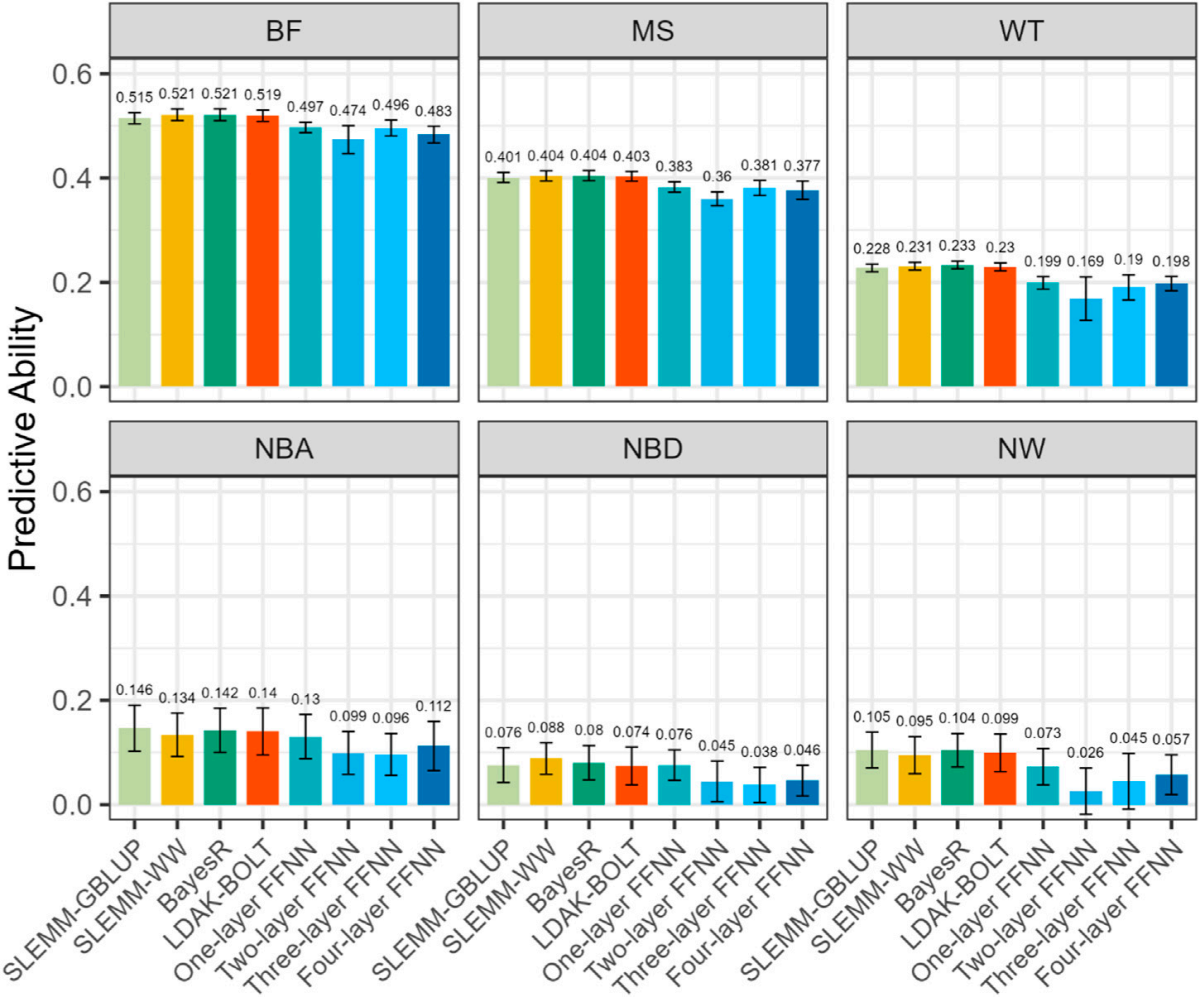
Predictive ability of genomic prediction methods for six quantitative traits. The predictive ability was measured as the Pearson correlation coefficient between observed and predicted phenotypic values in the test population across 20 iterations of repeated random subsampling validation. Error bars represent standard deviations.

We comprehensively evaluated the predictive abilities of eight genomic prediction methods for six complex traits in Duroc pigs, including three performance traits with moderate heritability (BF, MS, WT) and three reproduction traits with low heritability (NBA, NBD, NW). The scikit-learn’s ridge regression implementation achieved results largely same as GBLUP (Figure 3), consistent with their theoretical equivalence. The minor difference may arise from the 0.01 grid search step for heritability used to calculate the penalty value in ridge regression. It was therefore not included in the comparison of prediction accuracy in Figure 4. Although the predictive performance varied considerably across traits, several consistent patterns were observed in the relative performance of different methods.

The FFNN models consistently underperformed compared to conventional linear methods across all traits, despite being implemented with optimized hyper-parameters. Among the neural network architectures, the one-layer network yielded the best performance, achieving results comparable to GBLUP. This aligns with theoretical expectations, as a one-layer FFNN without activation function is mathematically equivalent to ridge regression, which in turn is equivalent to GBLUP under specific parameterizations. Meanwhile, the relationship between neural network complexity and predictive ability did not follow a consistent pattern. While the one-layer FFNN generally performed best among neural network models, the two-layer FFNN often showed the poorest performance, with three-layer and four-layer architectures achieving intermediate results for several traits.

Neural network methods also exhibited greater variability in predictive ability across validation iterations compared to conventional approaches. This inconsistency, combined with their lower average performance, indicates that despite their theoretical capacity to model complex relationships, FFNN models offered no practical advantage for genomic prediction of pigs in this study.

Among the linear methods evaluated, the differences in predictive performance were minimal. BayesR and SLEMM-WW demonstrated slightly superior predictive ability across most traits. There were also some distinct performance patterns across trait categories. For performance traits with moderate heritability (BF, MS, WT), linear methods accommodating more flexible genetic architectures demonstrated slightly superior predictive ability compared to GBLUP. For reproductive traits with low heritability (NBA, NBD, NW), this advantage diminished, with GBLUP achieving predictive abilities that matched or exceeded those of the more flexible methods.

### Computational speed

**FIGURE 5:**
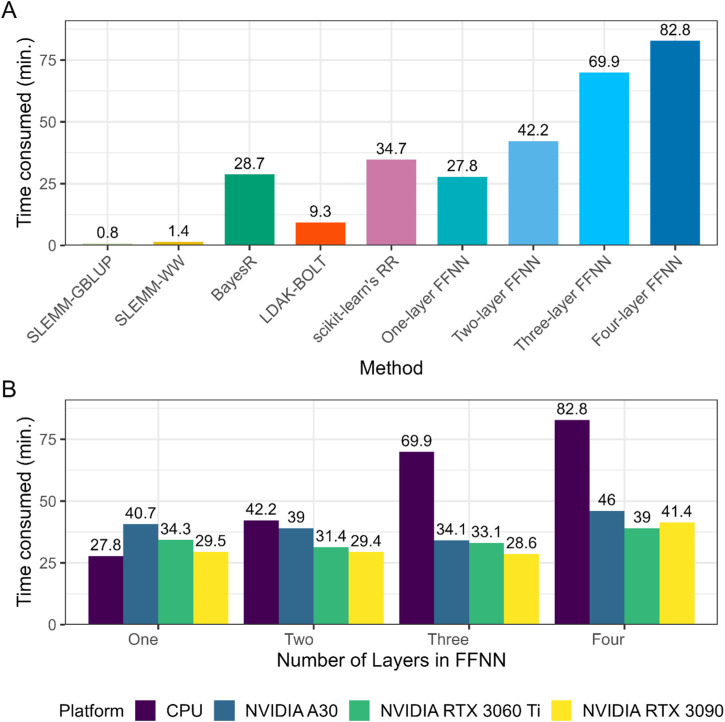
Computational efficiency of genomic prediction methods on different hardware platforms. All comparisons were based on one replicate from repeated random subsampling validation for BF. Genomic predictions were performed using 30,981 SNPs, with 21,385 individuals for training and 5,346 for testing. **(A)** Comparison of computational time (minutes) required for different genomic prediction methods implemented on CPU hardware. **(B)** Comparison of computational time (minutes) required for feed-forward neural networks implemented on different hardware platforms including CPU and three GPUs.

Computational efficiency is an important aspect for the practical application of genomic prediction methods in breeding programs. We first assessed the computational efficiency of all methods using the BF dataset on the CPU platform. Figure 5A shows the total computational time required for each method, including hyper-parameter tuning, model training, and prediction where applicable. Feed-forward neural networks were generally slower than most conventional linear models on the CPU platform. The computational cost was associated with deeper architectures and increased significantly with the increase of network depth. One-layer FFNN was the most efficient among the neural network implementations, and required similar computation time as BayesR. The computation time required for FFNNs exceeded all linear methods when using two or more layers. Among the linear methods, the two methods implemented in SLEMM software demonstrated superior computational efficiency. SLEMM-GBLUP completed the analysis in the shortest time, while SLEMM-WW required slightly longer due to the additional calculations for window-based weighting. LDAK-BOLT followed with moderate computational demands, and BayesR required substantially more time. The ridge regression implemented by scikit-learn exhibited the highest computational cost among linear methods, primarily due to the extensive grid search process for optimizing the regularization parameter.

The computational efficiency of feed-forward neural networks was further assessed using three GPU platforms: NVIDIA A30, NVIDIA RTX 3060 Ti, and NVIDIA RTX 3090 (Figure 5B). While for the one-layer structure (without hidden layers), the CPU implementation with 20 threads performed similarly to GPU implementations, highlighting that simpler linear models did not benefit from GPU acceleration, all neural network architectures with hidden layers demonstrated substantial computational acceleration when executed on GPU hardware. As model complexity increased with additional hidden layers, the computational advantage of GPU implementation became increasingly pronounced. The computational time on the CPU platform increased substantially with each additional layer, while the increase was considerably less dramatic on GPU platforms. For the four-layer neural network, GPU implementation reduced the computational time by approximately 2-fold compared to the CPU implementation. Differences among the three GPU platforms were relatively minor compared to the substantial gap between CPU and GPU performance for multi-layer networks. The high-end NVIDIA RTX 3090 provided the greatest computational acceleration, followed closely by the RTX 3060 Ti and A30. This performance advantage highlights the potential for GPU acceleration to mitigate the computational disadvantages of complex neural network architectures for genomic prediction. While neural networks remain more computationally intensive than the most efficient linear models even with GPU acceleration, the substantial reduction in processing time makes deeper architectures more viable for practical applications in genomic prediction pipelines.

## Discussion

In this study, we comprehensively evaluated FFNN models against conventional linear methods for pig genomic prediction. Performing genomic prediction using FFNN models was feasible for our pig dataset. With proper hyper-parameter tuning, these models can achieve reasonable predictive abilities, particularly the one-layer FFNN which performed closest to GBLUP. However, our results align with previous research showing neural networks provide no advantage and often perform worse than linear approaches for genomic prediction (Montesinos-López et al., 2021).

A primary difference between these methodologies lies in their modeling approach in that linear methods assume additive relationships between inputs and outputs, while neural networks, particularly those with hidden layers and non-linear activation functions, can theoretically capture complex, non-linear interactions among features. Several studies have reported that FFNN models can provide improvements in predictive ability when analyzing traits with substantial non-additive effects such as dominance and epistasis (Abdollahi-Arpanahi et al., 2020; Zingaretti et al., 2020; Costa et al., 2022; Perelygin et al., 2025). Notably, many research studies have reported that FFNN models achieved higher predictive accuracy compared to linear methods in plant genomic prediction research (Gianola et al., 2011; Khaki and Wang, 2019; Sandhu et al., 2021; Kelly and McLaughlin, 2024). Such performance advantages in plant species may derive from different breeding histories compared to livestock, potentially resulting in genetic architectures with greater preservation of non-additive genetic variance. However, most economically important traits in farm animals are influenced predominantly by additive genetic effects (Jiang et al., 2017; Vitezica et al., 2018; de Oliveira et al., 2023). Although non-additive genetic effects may have more pronounced influence on reproductive traits (Jiang et al., 2017), the limited sample size in our study may have restricted the ability of neural networks to capture and leverage these effects effectively. Moreover, the theoretical advantage of neural networks in capturing non-linear relationships may be diminished when conventional methods are properly specified to incorporate these effects. Linear models with appropriate parameterization to account for non-additive effects can match or exceed the predictive performance of deep learning methods when modeling traits with strong non-additive architectures (Zingaretti et al., 2020).

Determining optimal hyper-parameters represents a significant challenge in neural network implementation for genomic prediction. Neural networks, unlike linear methods, operate within a vast hyper-parameter space that substantially influences model performance. Hyper-parameters that work well for one trait or population may not generalize to others (Zingaretti et al., 2020), necessitating trait-specific optimization that further increases the computational burden. Traditional grid search approaches become practically impossible for complex neural networks due to the exponential growth of the search space as the number of hyper-parameters increases. Random search offers some relief by sampling hyper-parameter combinations randomly, but it still requires substantial computational resources and may not efficiently explore the most promising regions of the search space (Bergstra and Bengio, 2012). To alleviate this problem, we applied hyperband tuning (Li et al., 2018), an efficient multi-fidelity hyper-parameter optimization strategy that evaluates numerous configurations with limited resources and progressively allocates more computational time only to promising candidates. Other efficient approaches such as Bayesian optimization methods (Snoek et al., 2012; Waldmann et al., 2020) and evolutionary algorithms (Storn and Price, 1997; Han et al., 2021) have also been explored in deep learning and genomic prediction contexts.

Neural networks also differ fundamentally from linear methods in how they process data during training. Linear methods typically process all individuals simultaneously, while neural networks utilize batch processing, where only a subset of data is processed in each training iteration. The choice of batch size in this context introduces a trade-off between computational efficiency and model performance. Larger batches provide more stable gradient estimates and better hardware utilization but require more memory, while smaller batches offer better generalization in some cases but slower convergence (Goodfellow, 2016). Excessively large batch sizes can exceed GPU memory capacity, particularly for multi-layer architectures where parameter counts increase substantially. Therefore, we determined the batch size before proceeding with the main hyper-parameter tuning process.

The hyper-parameters optimized in this study directly impact how neural networks model the relationship between genotypes and phenotypes and are critical to overall model performance. The number of neurons determines the model’s capacity to capture complex patterns. More neurons can capture more complex genetic relationships but risk modeling noise. Activation functions (ReLU, sigmoid, tanh, softmax) determined the non-linear transformations applied throughout the network. While ReLU is commonly used in deep learning due to its simplicity and computational efficiency (Glorot et al., 2011), the optimal choice varies by trait and architecture rather than showing universal superiority (Montesinos-López et al., 2021). The learning rate controls how quickly the neural network updates its weights during training, balancing between convergence speed and training stability. L2 regularization and dropout mitigate overfitting by penalizing large weights and randomly deactivating neurons, respectively. Excessive regularization or dropout can lead to underfitting by impeding the network’s learning capacity (Srivastava et al., 2014; Goodfellow, 2016).

Our study employed repeated random subsampling validation with the same 20 random 80/20 splits to evaluate all methods, ensuring fair comparison with no methodological bias. Each split maintained strict separation between training and testing sets with no data leakage. While alternative validation strategies such as k-fold cross-validation or year-based validation could provide different perspectives on absolute performance values, they would yield similar relative comparisons among methods when applied consistently. Our repeated random subsampling approach is methodologically sound and well-suited for our research objectives of comparing method performance within this population. The 80/20 split ratio provides a good balance between training data availability and test set size for reliable evaluation.

While differences in predictive ability among methods were modest, computational efficiency varied substantially. The two methods implemented in SLEMM demonstrated exceptional speed on CPU hardware, completing analyses in under 2 min for genomic prediction with approximately 20,000 individuals. In comparison, FFNN models required considerably more computational resources on the same platform, with processing time increasing significantly as network complexity increased. However, when training was performed on a GPU, the additional time required for deeper architectures was minimal. This difference can be attributed to the inherent design of GPUs, which are specialized for parallel processing and are capable of performing many calculations simultaneously. Unlike CPUs that process operations sequentially, GPUs can simultaneously execute multiple matrix and vector operations fundamental to neural network training (Oh and Jung, 2004). Consequently, GPU acceleration significantly reduces training times for deep neural networks, making these computationally intensive models practical for large-scale genomic prediction. The relative performance among the three tested GPU platforms (NVIDIA A30, RTX 3060 Ti, and RTX 3090) showed only minor differences, suggesting that even consumer-grade GPUs can provide significant acceleration for genomic prediction applications. Although the computational requirements for neural networks with GPU acceleration remain substantially higher than those of optimized linear methods, this hardware accessibility creates opportunities to investigate more complex neural network architectures that may better capture non-additive genetic effects and improve prediction accuracy for traits with complex inheritance patterns.Therefore, for practical pig breeding applications, conventional linear methods, particularly SLEMM-WW, currently offer the optimal balance of prediction accuracy and computational efficiency. FFNN models demonstrate no advantage compared to conventional methods as they demand more computational resources while typically yielding lower predictive ability. However, neural network approaches remain promising for future applications beyond the numeric phenotypes used in this research. For instance, when phenotypic data takes the form of images or videos (Billah et al., 2025; Xu et al., 2025), neural networks may offer substantial advantages due to their established performance in visual data processing. Additionally, alternative neural network architectures specifically designed for genomic prediction merit exploration. Convolutional neural networks that capture local patterns across the genome, recurrent neural networks that model sequential dependencies, or transformer models that can identify complex relationships between distant genomic regions may prove more effective than standard feed-forward architectures. Furthermore, incorporating biological information, such as gene annotations or pathway data, to guide neural network design might improve their performance by focusing the model on biologically plausible relationships (Fortelny and Bock, 2020). These potential directions highlight the need for continued research in applying machine learning approaches to genomic prediction.

## Conclusion

In summary, we comprehensively evaluated FFNN models for genomic prediction in Duroc pigs. Despite extensive hyper-parameter optimization, neural networks consistently underperformed all conventional linear methods across all traits examined. While GPU acceleration significantly reduced computational time for multi-layer networks, the combination of lower predictive ability and higher computational demands makes complex neural networks less practical than optimized linear methods for routine genomic prediction in commercial pig breeding programs. Although neural networks have transformed many fields of artificial intelligence, their application to genomic prediction requires careful consideration of trait genetic architecture, sample size, and computational constraints. Our findings suggest that for current pig breeding applications, conventional linear methods remain the most effective approach, and among linear methods we tested, SLEMM-WW offers the optimal balance of computational efficiency and predictive ability.

## Data Availability

The data analyzed in this study is subject to the following licenses/restrictions: All the data used in this study are the property of Smithfield Premium Genetics (Rose Hill, NC, United States). Restrictions apply to the availability of these data, which were used under license for the current study and are not publicly available. Requests to access these datasets should be directed to Kent Gray, kgray@smithfield.com.
